# TNFα priming through its interaction with TNFR2 enhances endothelial progenitor cell immunosuppressive effect: new hope for their widespread clinical application

**DOI:** 10.1186/s12964-020-00683-x

**Published:** 2021-01-04

**Authors:** Mahsa Nouri Barkestani, Sara Shamdani, Mazdak Afshar Bakshloo, Nassim Arouche, Bijan Bambai, Georges Uzan, Sina Naserian

**Affiliations:** 1 INSERM UMR-S-MD 1197, Hôpital Paul Brousse, Villejuif, France; 2 Paris-Saclay University, Villejuif, France; 3 National Institute for Genetic Engineering and Biotechnology (NIGEB), Tehran, Iran; 4 CellMedEx, Saint Maur Des Fossés, France

**Keywords:** Endothelial progenitor cells (EPCs), Endothelial colony forming cells (ECFCs), T cells, Immunomodulation, TNFα-TNFR2 signaling pathway, TNFα priming

## Abstract

**Background:**

Bone marrow derived endothelial progenitor cells (EPCs) are immature endothelial cells (ECs) involved in neo-angiogenesis and endothelial homeostasis and are considered as a circulating reservoir for endothelial repair. Many studies showed that EPCs from patients with cardiovascular pathologies are impaired and insufficient; hence, allogenic sources of EPCs from adult or cord blood are considered as good choices for cell therapy applications. However, allogenic condition increases the chance of immune rejection, especially by T cells, before exerting the desired regenerative functions. TNFα is one of the main mediators of EPC activation that recognizes two distinct receptors, TNFR1 and TNFR2. We have recently reported that human EPCs are immunosuppressive and this effect was TNFα-TNFR2 dependent. Here, we aimed to investigate if an adequate TNFα pre-conditioning could increase TNFR2 expression and prime EPCs towards more immunoregulatory functions.

**Methods:**

EPCs were pre-treated with several doses of TNFα to find the proper dose to up-regulate TNFR2 while keeping the TNFR1 expression stable. Then, co-cultures of human EPCs and human T cells were performed to assess whether TNFα priming would increase EPC immunosuppressive and immunomodulatory effect.

**Results:**

Treating EPCs with 1 ng/ml TNFα significantly up-regulated TNFR2 expression without unrestrained increase of TNFR1 and other endothelial injury markers. Moreover, TNFα priming through its interaction with TNFR2 remarkably enhanced EPC immunosuppressive and anti-inflammatory effects. Conversely, blocking TNFR2 using anti-TNFR2 mAb followed by 1 ng/ml of TNFα treatment led to the TNFα-TNFR1 interaction and polarized EPCs towards pro-inflammatory and immunogenic functions.

**Conclusions:**

We report for the first time the crucial impact of inflammation notably the TNFα-TNFR signaling pathway on EPC immunological function. Our work unveils the pro-inflammatory role of the TNFα-TNFR1 axis and, inversely the anti-inflammatory implication of the TNFα-TNFR2 axis in EPC immunoregulatory functions. Priming EPCs with 1 ng/ml of TNFα prior to their administration could boost them toward a more immunosuppressive phenotype. This could potentially lead to EPCs’ longer presence in vivo after their allogenic administration resulting in their better contribution to angiogenesis and vascular regeneration.

**Video Abstract**

## Background

Several cardiovascular disorders (CVD) cause endothelial cell damage, disrupting vascular homeostasis leading to various complications [1]. Several innovative therapies, mostly centered on restoring blood supply via gene therapy to induce the pro-angiogenic growth factor or direct administration of pro-angiogenic mediators like vascular endothelial growth factor (VEGF), basic fibroblast growth factor (bFGF), hepatocyte growth factor (HGF) and hypoxia-inducible factor 1-alpha (HIF-1), have been used to achieve this goal without very promising results [2–6]. However, stem cell therapy predominantly based on mesenchymal stem cells (MSCs) and endothelial progenitor cell (EPCs) or a mixture of both, is recently proved to be an effective approach to stimulate angiogenesis/vasculogenesis both in-vitro and in-vivo [7, 8]. Accordingly, we have recently reported a successful administration of autologous EPCs for the treatment of right ventricle (RV) failure in a piglet model of chronic thromboembolic pulmonary hypertension (CTEPH) [9].

Bone marrow (BM) derived circulating progenitors for the endothelial lineage, called EPCs, are non-differentiated and immature endothelial cells (ECs) first isolated from adult blood by Asahara [10]. These cells are capable of homing to vascular structures while they differentiate into mature ECs [11, 12], and contribute in endothelium homeostasis and maintenance of vascular integrity [13–15]. In-vitro, Endothelial Colony Forming Cells (ECFCs) are distinct from other EPCs. ECFCs can be also isolated from umbilical cord blood (CB-ECFCs) [16]. We have already reported that CB-ECFCs give rise to higher number of colonies and can be extensively expanded compared to ECFCs derived from adult peripheral blood (APB-ECFCs) which reach to state of cellular senescence after few passages [17–19]. They can incorporate into vascular network and form stable vessels [20–22]. ECFCs express all EC markers, notably CD31, CD144 and KDR (VEGFR2), in addition to CD34 and CD133 hematopoietic markers and they are negative for CD45 and CD14 common leukocyte antigen and monocytes markers respectively. In comparison to mature ECs, ECFCs bear features of stem/progenitor cells such as high clonogenicity and proliferation rate [23, 24], stemness gene expression and enhanced reprogramming efficiency into induced pluripotent stem cells [25]. Moreover, after proper external stimuli, they could gain specialized EC properties such as brain microvascular or arterial ECs [26]. Despite our knowledge to efficiently isolate and expand them ex-vivo, no specific marker has been attributed to these cells to distinguish them from the rest of ECs. However, stressing on stem cell features, our team has reported that ECFCs have some unique immunological properties that could functionally discriminate them from other ECs. We have recently showed that in a complete contrast to mature human aortic endothelial cells (HAEC), both CB-ECFCs and APB-ECFCs are immunosuppressive and can efficiently down-modulate the immune response [27–29].

Inflammatory environment is essential for EC migration and angiogenic function [30]. Tumor necrosis factor alpha (TNFα) is a pro-inflammatory cytokine that could regulate both pro- and anti-angiogenic activities [31–33]. The concentration of TNFα and duration of exposure can control this dual effect [34]. TNFα recognizes two distinct transmembrane receptors, TNFR1/P55 and TNFR2/P75. TNFR1 is ubiquitously expressed on all cell types and its binding with TNFα provokes cell death and apoptosis mechanisms. However, TNFR2 is expressed on limited cells like ECs, immune cells, neural cells and MSCs and its interaction with TNFα leads to cell survival, activation and proliferation [35–37]. It was reported that TNFα could increase the expression of pro-angiogenic mediators such as VEGF, bFGF, and interleukin-8 (IL-8) in ECs [38–40]. Unlike TNFα-TNFR1 signaling pathway which is involved in different deleterious mechanisms such as increased inflammation and tissue damage in myocardial ischemic injuries and toxic effect in myocardial infraction [41, 42], TNFα-TNFR2 signaling supports pro-angiogenic and regenerative mechanisms like protective effect in graft versus host disease (GVHD) [43, 44], adult infract myocardium [42], heart ischemic injuries [45] and aging [46, 47]. Accordingly, further in-vivo studies demonstrated that many critical ECFC physiological factors like cell survival, mobilization, differentiation and functional aspects like VEGF production and ischemia-induced collateral vessel development depend on TNFα-TNFR2 signaling pathway [47]. Likewise, specific transgenesis of TNFR2 on ECs led to a significant promotion in arteriogenesis and angiogenesis, a phenomenon that was not observed in TNFR2 KO mice [48].

Nevertheless, our knowledge regarding the effect of inflammatory micro-environment on EFCF immunological properties is still very poor. We have recently demonstrated that human ECFCs are efficiently capable of inducing new functional vessels in xenogeneic ischemic immunocompetent mice and are immunotolerated by the mouse immune system for several days after their first administration [29]. Furthermore, to investigate the mechanism behind their immunomodulatory function, we assessed the immunosuppressive effect of CB-ECFCs and APB-ECFCs on mouse T cells. We showed that ECFCs not only are able to suppress different T cell populations in a dose dependent manner but are able to decrease the activation and pro-inflammatory cytokine production profile of both CD4 and CD8 conventional T cells (T convs) [27, 28]. Various previous studies have demonstrated a direct relation between the expression of TNFR2 and certain level of immunoregulatory effect [44, 49, 50]. In order to assess whether the expression of TNFR2 on ECFCs is also related to their immunoregulatory functions; we have blocked the TNFα-TNFR2 axis and demonstrated that this signaling pathway is in complete control of ECFC immunosuppressive effect [28].

Observing the immunomodulatory role of TNFR2 and the importance of TNFα in ECFC biology, we sought to investigate whether a pre-treatment of ECFCs with TNFα could prime them towards more anti-inflammatory phenotypes. Hence, we first evaluated the proper dose of TNFα, in which ECFCs (1) have the highest increase of TNFR2 but stable TNFR1 expression, and (2) correctly enter inflammatory status without crossing the threshold of being injured and apoptotic. Thereafter, we primed ECFCs from different sources (cord and adult blood) and assessed whether the ECFC immunosuppressive and immunomodulatory effect will be boosted against human HLA mismatched allogenic T cells.

## Methods

### ECFC and T cell isolation

CB samples from healthy full term newborns were obtained from the CB Bank of St Louis Hospital (Paris, France). Human APB from healthy adults was obtained from the French Establishment of Blood (EFS) (Rungis, France). Mononuclear cells (MNCs) were obtained by density gradient centrifugation using Pancoll human solution (Pan-Biotech).

For ECFC isolation, MNCs were seeded into rat-tail collagen type-I (BD-Bioscience) coated wells as previously described [26]. ECFC colonies appeared after 7–20 days of culture. From passage 1 (P1), cells were seeded at 5000 cells/cm^2^ and grew in EGM-2MV medium (Lonza).

Human pan T cell isolation kit (Miltenyi-Biotec) was used to isolate total CD3^+^ T cells from MNCs of peripheral adult blood. Furthermore, CD25^+^ cells were depleted from the CD3^+^ T cell population using anti-CD25 biotin conjugated antibody (Miltenyi-Biotec), followed by anti-biotin microbeads staining (Miltenyi-Biotec). Then, the magnetic-activated cell sorting (MACS) method was used in all cell isolations. The resulting CD3^+^CD25^−^ T cells, more than 93% purity, were cultured in the presence of ECFCs. The isolation of T cells from co-culture in presence of ECs is based on the biological capacity of ECs to adhere to plastic plates, and T cells that stay in suspension; hence, they were collected with gentle resuspension and aspiration.

### Co-culture of ECs and T cells

Human CB-ECFCs (P3 to P7) or ABP-ECFCs (P3 to P5) were seeded into 6 or 12 well plates (Falcon) and incubated at least for 3 h in EGM2 medium. Then, freshly isolated human T cells were added to ECFCs at different doses depending on experimental conditions in RPMI medium (containing 10% FBS, 1% HEPES buffer, 5 × 10^−5^ M β-mercaptoethanol and 1% penicillin/streptomycin/neomycin (Gibco)). All co-culture and control experiments were performed in 50% EGM2 and 50% RPMI media at 37 °C in 5% CO_2_.

To prime ECFCs, we pre-treated them for 24 h (day -1) with 0.01, 0.1, 1, 10, 50 and 100 ng/ml of premium grade recombinant-human-TNFα (Miltenyi-Biotec). In order to block TNFR2, we used 2 µg/ml human-TNFR2/CD120b/TNFRSF1B neutralizing antibody (Sino Biological), 24 h prior TNFα addition (day -2).

### T cell proliferation assay

3 × 10^4^ ECs (APB- ECFCs, CB- ECFCs) were co-cultured with 6 increasing doses of human CD3^+^CD25^−^responder T cells in a total volume of 1 ml. The doses were 1/1, 1/2, 1/4, 1/8, 1/16, 1/32 (ECFCs/T cells). T cells were stained with carboxyfluorescein succinimidyl ester (CFSE) (Invitrogen™) and polyclonaly stimulated by Dynabeads human T-activator CD3/CD28 according to supplier’s protocol (Gibco). 1 × 10^5^ CFSE labelled, activated or non-activated T cells alone were used as controls. After 3 days, T cells were collected and immunostained and the percentage of proliferating cells among CD4^+^ and CD8^+^ T cells was analysed by flow cytometric measurements. Divided cells were identified by the decrease in CFSE expression due to its dilution after each division. Events acquired on a LSRFORTESSA flow cytometer (BD-Biosciences) and analyzed using FlowJo software v10 (FlowJo-LLC).

### T lymphocytes activation assay

3 × 10^4^ CB or APB-ECFCs were seeded in 12-well plates and co-cultured with 1.8 × 10^5^ activated human T cells (1/6 ECFC/T cell, fixed intermediate ratio) in a total volume of 1 ml. 1.8 × 10^5^ activated and non-activated human T cells were used as controls. After 3 days, T cells were collected by gentle aspiration and immunostained with mixes of following antibodies: VioBlue-anti-CD4, Biotin-anti-CD8 or PE-Vio770-anti-CD8, APC-anti-GITR, Biotin-anti-CD25 or PE-anti-CD25, PE-Vio770-anti-ICOS, PE-anti-TNFR2 or FITC-anti-TNFR2 (Miltenyi-Biotec) and Streptavidin-PE-Cy5 (eBioscience) antibodies (Abs). Events acquired on a LSRFORTESSA flow cytometer (BD-Biosciences) and analyzed using FlowJo software v10 (FlowJo-LLC).

### Apoptosis measurement assay

CB-ECFCs were pre-treated for 24 h with 0.01, 0.1, 1, 10, 50 and 100 ng/ml of TNFα. Untreated CB-ECFCs from the same donors and passages were used as control group. Then, cells were detached using cell dissociation reagent TrypLE (Gibco). Percentage of apoptotic cells were evaluated after immunostaining by annexin V and popidium iodide (PI) antibodies using Annexin V-FITC kit (Miltenyi-Biotec) according to the supplier’s protocol. Events acquired on a LSRFORTESSA flow cytometer (BD-Biosciences) and analyzed using FlowJo software v10 (FlowJo-LLC).

### Endothelial cell marker measurement

CB-ECFCs were pre-treated for 24 h with 0.01, 0.1, 1, 10, 50 and 100 ng/ml of TNFα. Untreated CB-ECFCs from the same donors and passages were used as control group. Then, cells were detached using cell dissociation reagent TrypLE (Gibco) and proceeded to immunostaining using mixes of following antibodies: FITC-anti-CD31, VioBlue-anti-CD144, PE-Vio770-anti-VEGFR2 (KDR), APC-anti-TNFR1, PE-anti-TNFR2, APC-anti-ICAM (CD54), PE-anti-VCAM (CD106), APC-anti-TIE2 (CD202b), REA control (s)-APC, REA control (s)-PE (Miltenyi-Biotec). Events acquired on a LSRFORTESSA flow cytometer (BD-Biosciences) and analyzed using FlowJo software v10 (FlowJo-LLC).

### Statistical analysis

Prism (GraphPad) was used for statistical analysis. Shapiro–Wilk normality test was performed to assess the normal distribution of data. Student *t* test or one-way ANOVA with post hoc analysis was performed depending on the number of comparatives. For cytometry analysis, we have normalized the MFI values with T-cell alone control group. Then we used unpaired, two-tailed Student *t* tests or one way ANOVA for *P* value generation.

## Results

### Pre-treatment of ECFCs with 1 ng/ml of TNFα enhances TNFR2 expression

**Fig. 1:**
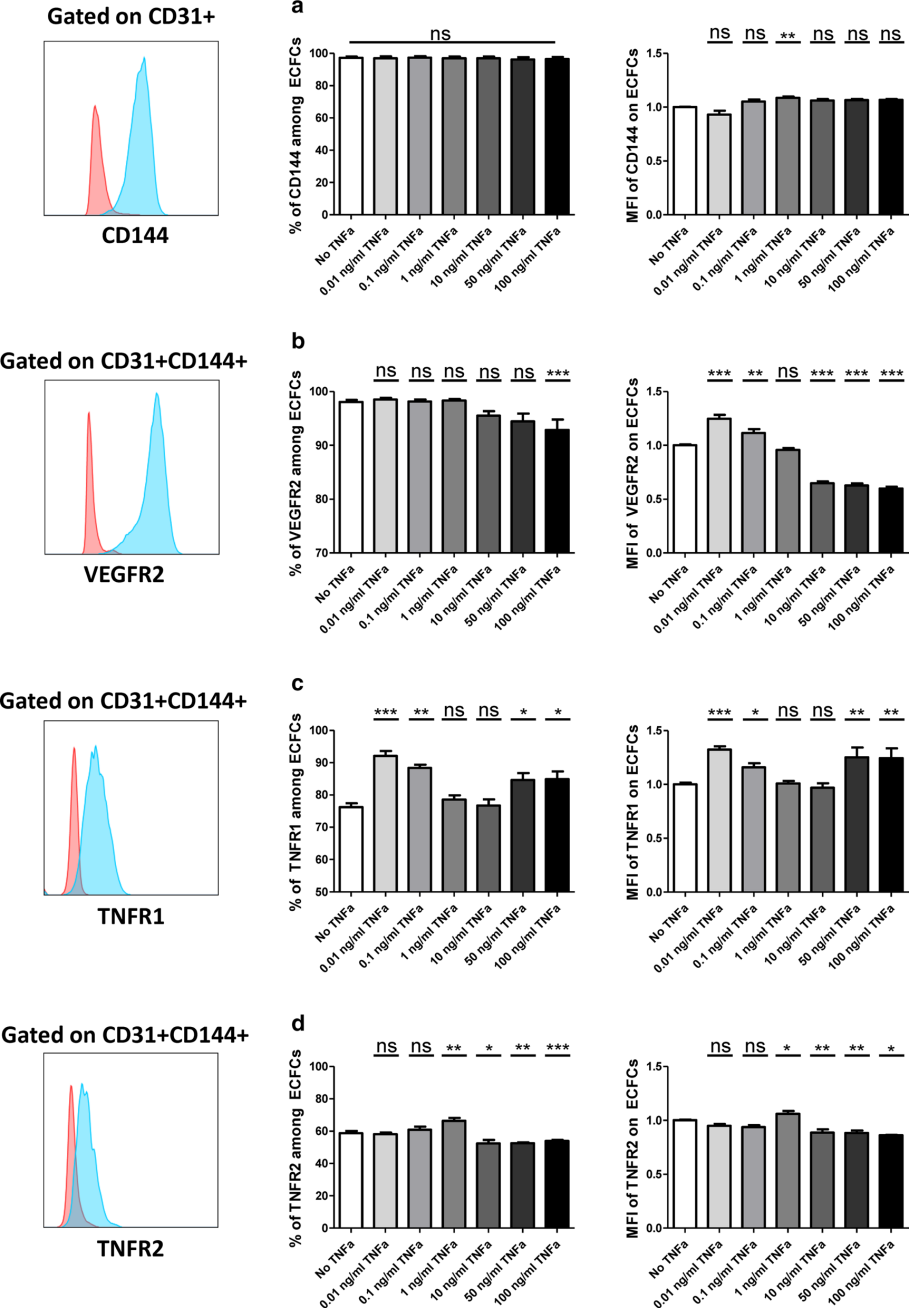
The impact of TNFα treatment on endothelial markers. CB-ECFCs were treated with different TNFα doses for 24 h and assessed for the percentage of expression and the mean fluorescent intensity of their surface markers. **a** The expression of CD144 among total CD31^+^ cells (n = 14), **b** the expression of VEGFR2 among CD31^+^CD144^+^ cells (n = 18), **c** the expression of TNFR1 among CD31^+^CD144^+^ cells (n = 20), **d** the expression of TNFR2 among CD31^+^CD144^+^ cells (n = 20). In representative flow cytometry panels, red histograms depict isotype controls and blue histograms depict the positive expression of desired markers. Data are represented as mean value ± SEM from 4 independent experiments. One way ANOVA analysis was performed to generate *P* values. ns: non-significant, *P < .05; **P < .01; ***P < .001

We first investigated if treating ECFCs with TNFα could change the expression of ECFC principle markers. Therefore, CB-ECFCs were incubated with increasing doses of TNFα (0, 0.01, 0.1, 1, 10, 50, 100 ng/ml). After 24 h, no difference was noticed in CD31 expression (data not shown). The same result was observed for the percentage of CD144 expression; however, we detected a slight increase in CD144 expression level (Mean Fluoresce Intensity (MFI)) starting from 0.1 ng/ml of TNFα which was significant only with 1 ng/ml treatment (Fig. 1a). In case of VEGFR2, we observed no difference in the percentage of VEGFR2 expression until 1 ng/ml of TNFα but a dose dependent decrease in higher doses. The MFI of VEGFR2 was increased with 0.01 and 0.1 ng/ml of TNFα then reached to basal level in 1 ng/ml and significantly dropped in higher doses (Fig. 1b).

We then evaluated the impact of TNFα stimulation on the expression of TNFRs. The percentage of expression and MFI of TNFR1 was dramatically increased when ECFCs were treated with low doses of TNFα (0.01 and 0.1 ng/ml) and then reached to basal level in intermediate doses of 1 and 10 ng/ml and elevated again following high doses of 50 and 100 ng/ml of TNFα treatment (Fig. 1c). Interestingly, while 0.01 and 0.1 ng/ml of TNFα did not changes the TNFR2 expression, pre-treating ECFCs with 1 ng/ml significantly increased the percentage of expression and MFI of TNFR2. The higher TNFα doses significantly reduced the TNFR2 expression (Fig. 1d).

This proved that pre-treatment of ECFCs with 1 ng/ml of TNFα can increase the TNFR2 expression without a dramatic change in the basal expression level of CD144, VEGFR2, and pro-inflammatory TNFR1 marker.

### Pre-treatment of ECFCs with 1 ng/ml of TNFα provides sufficient inflammation

**Fig. 2:**
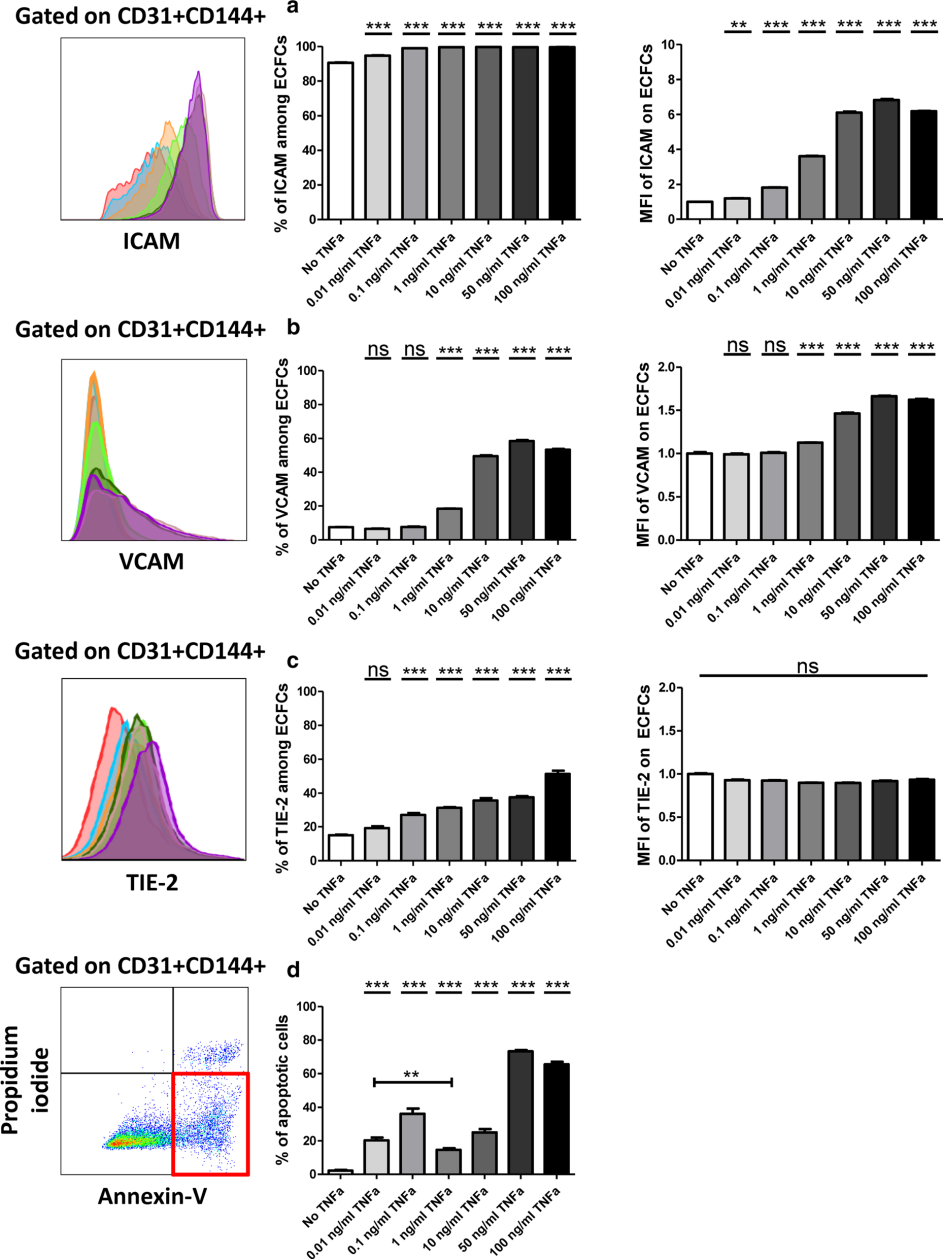
The impact of TNFα treatment on endothelial inflammatory/injury markers. CB-ECFCs were treated with different TNFα doses for 24 h and assessed for the percentage of expression and the mean fluorescent intensity of their surface markers. **a** The expression of ICAM among total CD31^+^CD144^+^ cells (n = 8), **b** the expression of VCAM among CD31^+^CD144^+^ cells (n = 8), **c** the expression of TIE-2 among CD31^+^CD144^+^ cells (n = 8), **d** the percentage of annexin^+^PI^−^ apoptotic cells among CD31^+^CD144^+^ cells (n = 8). In representative flow cytometry panels, red histograms depict the expression of desired marker on untreated ECFCs. The other colourful histograms depict the expression of desired marker on ECFCs after treating with the following doses of TNFα: blue: 0.01 ng/ml; orange: 0.1 ng/ml; light green: 1 ng/ml; dark green: 10 ng/ml; pink: 50 ng/ml; purple: 100 ng/ml. Representative flow cytometry dot plots show the percentage of apoptotic ECFCs after 1 ng/ml of TNFα treating. Data are represented as mean value ± SEM from 2 independent experiments. One way ANOVA or Student *t* test analysis was performed to generate *P* values. ns: non-significant, *P < .05; **P < .01; ***P < .001

One of the main aims of this study was to achieve a controlled inflammatory environment in which ECFCs up-regulate TNFR2 expression without passing the borderline of toxic and pro-apoptotic state. CB-ECFCs were incubated with increasing doses of TNFα. After 24 h, cells were analyzed for the expression of ICAM, VCAM and TIE-2 pro-inflammatory/injury markers [51]. We observed that 0.01 and 0.1 ng/ml of TNFα provided low inflammation. In these setting, ICAM and TIE-2 (only with 0.1 ng/ml) markers were up-regulated while VCAM remained untouched (Fig. 2a–c). Nevertheless, this was accompanied by a significant increase in the percentage of apoptotic CD31^+^CD144^+^ cells (20.26% and 36.11% respectively) (Fig. 2d). Interestingly, our data showed that 1 ng/ml of TNFα was the threshold between the low inflammatory and extensive inflammatory/injury status. In this dose, we observed an intermediate expression of ICAM, VCAM and TIE-2 markers (Fig. 2a–c) and remarkably lower apoptosis (14.63%) compared to lower doses of TNFα (Fig. 2d). The higher doses of TNFα (10, 50 and 100 ng/ml) entered ECFCs into uncontrolled and toxic inflammation with considerably higher ICAM, VCAM and TIE-2 expression. This was accompanied by high levels of apoptosis notably with 50 and 100 ng/ml of TNFα (25.03%, 73.3% and 65.54% respectively). Therefore, 1 ng/ml of TNFα was validated as the ideal dose to treat ECFCs prior co-culturing with T cells.

### TNFα priming increases ECFC immunosuppressive effect

**Fig. 3:**
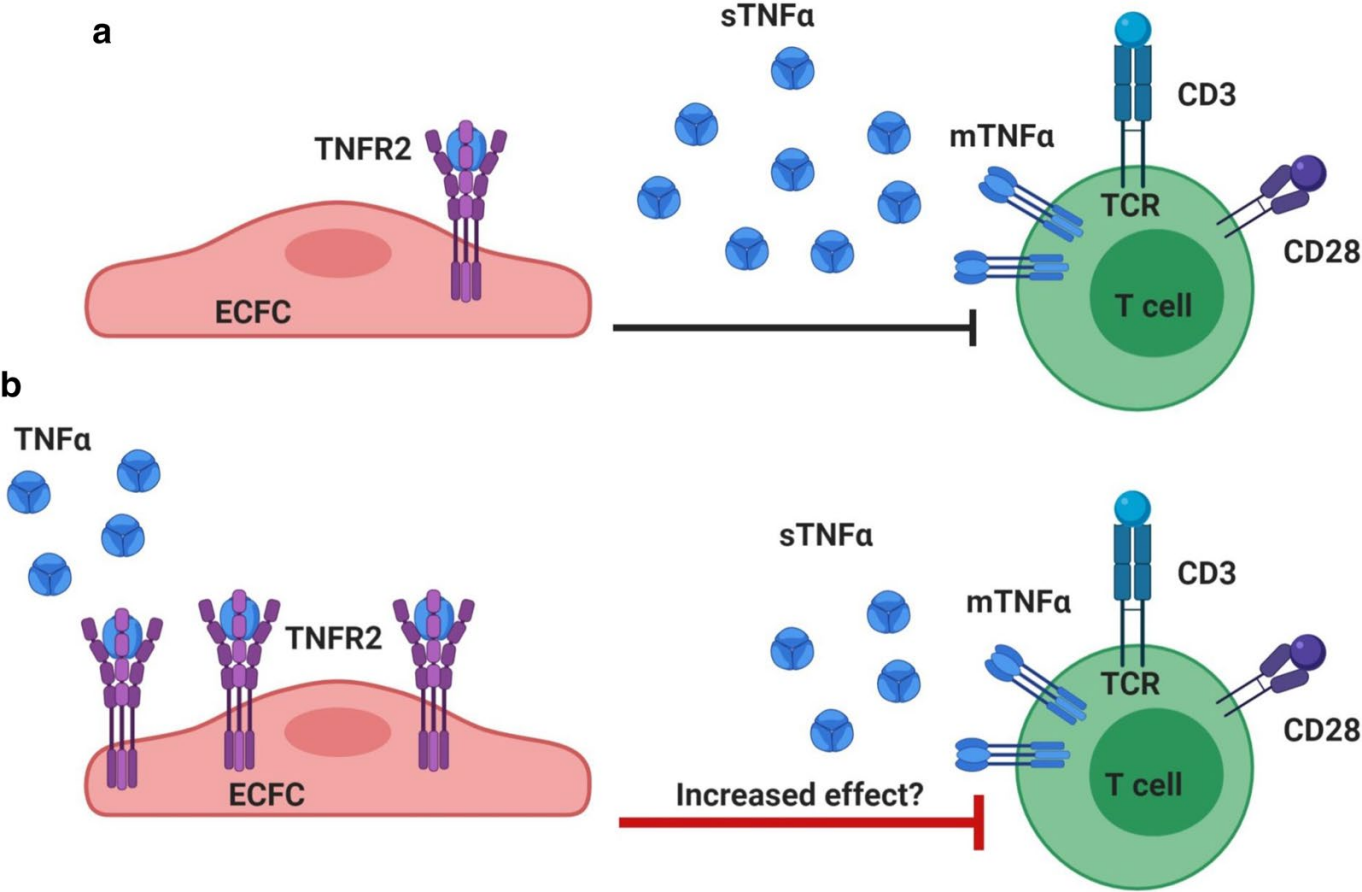
Hypothetic interaction between ECFCs and T cells. **a** This schematic depicts the involvement of TNFα-TNFR2 axis in immunosuppressive effect of ECFCs on T cells. Briefly, T cells upon activation produce pro-inflammatory cytokines including TNFα. This cytokine through its interaction with TNFR2 mediates ECFC immunosuppressive function. **b** This schematic depicts our hypothesis based on the priming effect of TNFα treatment on ECFC immunosuppressive effect. Briefly, pre-treating ECFCs with proper dose of TNFα could increase the expression of TNFR2 which in turn boosts ECFCs toward enhanced immunosuppressive and immunomodulatory functions

We previously demonstrated that ECFCs can suppress T cells and this effect was dependent on the production of TNFα by activated T cells and the expression of TNFR2 by ECFCs (Fig. 3a) [27, 28]. Here, we aimed to explore if the pre-treatment of ECFCs with TNFα could boost TNFR2 expression and consequently their immunosuppressive effect (Fig. 3b).

**Fig. 4:**
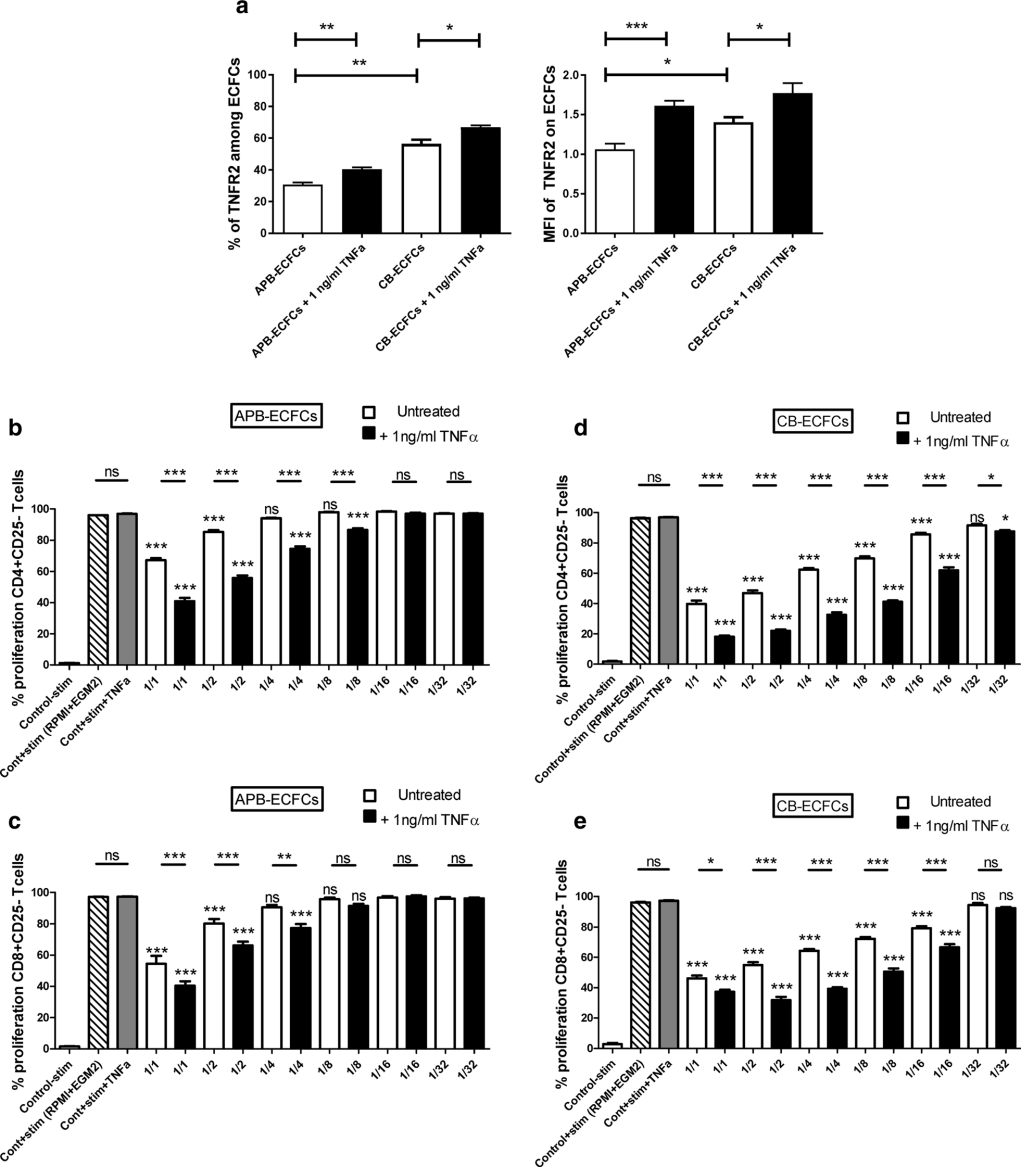
TNFα priming enhances ECFC immunosuppressive effect. ECFCs were treated with 1 ng/ml of TNFα for 24 h and **a** randomly assessed for the expression of TNFR2 markers (n = 5). Activated CFSE^+^CD3^+^CD25^−^ responder T cells were co-cultured with (**b**, **c**) APB-ECFCs (n = 8) and (**d**, **e**) CB-ECFCs in different ECFC/T cell ratios (n = 12). Proliferation of CD4^+^ T cells (**b**, **d**) and CD8^+^ T cells (**c**, **e**) was measured by flow cytometry. The first bar represents the unstimulated T cells alone, the second bar represents the anti-CD3/CD28 stimulated T cells alone in 50% RPMI + 50% EGM2 media, while the third bar is the anti-CD3/CD28 stimulated T cells alone in 50% RPMI + 50% EGM2 media + 1 ng/ml TNFα. Data are represented as mean value ± SEM collected from 3 different experiments. One way ANOVA or Student *t* test analysis was performed to generate *P* values. ns: non-significant, *P < .05; **P < .01; ***P < .001. Stim: Anti-CD3/CD28 activation Beads

We treated two sources of ECFCs (CB and APB-ECFCs) with 1 ng/ml of TNFα for duration of 24 h. As expected, this treatment effectively increased the percentage and MFI of TNFR2 on both types of ECFCs (Fig. 4a). Then, untreated or primed ECFCs were co-cultured with CFSE labeled human CD3^+^CD25^−^ responder T cells in 6 increasing ratios (1/1 to 1/32 for ECFCs/T cells). CD25^+^ T cells were depleted from starting T cell population to eliminate (1) pre-activated T cells and (2) unspecific immunosuppression by natural CD25^high^ Tregs. After 3 days, T cells were collected and the proliferation capacity of CD4^+^ and CD8^+^ T cells was measured. To observe the effect of EGM2 medium and the probable TNFα remnant on T cells, two control group were considered in which T cells were cultured either in 50% EGM2 + 50% RPMI or 50% EGM2 + 50% RPMI + 1 ng/ml of TNFα. No difference was observed between those controls (Fig. 4b–e). As expected, we observed a significant dose dependent decrease in proliferation capacity of both CD4^+^ and CD8^+^ T cells when co-cultured with APB-ECFCs (Fig. 4b, c) and CB-ECFCs (Fig. 4d, e). Among untreated APB-ECFCs, the immunosuppressive effect was only observed in 1/1 and 1/2 ratios (30.65% and 12% of suppression, respectively) for CD4^+^ T cells and equally for CD8^+^ T cells (43.87% and 17.36% of suppression, respectively) and then was lost for more elevated ratios (Fig. 4b, c). Interestingly, pre-treating with 1 ng/ml TNFα increased APB-ECFC immunosuppressive capacity up to 1/8 ratio with 10.75% of CD4^+^ T cell suppression versus 3% in 1/8 untreated condition (Fig. 4b) and a tendency to more CD8^+^ T cell immunosuppression in 1/8 ratio (1.37% in untreated versus 5.75% in primed condition) (Fig. 4c). A stronger immunosuppression of T cells was observed after co-culturing with untreated CB-ECFCs, starting from 1/1 (59.12% of suppression) up to 1/16 ratio (11.78% of suppression) for CD4^+^ T cells and from 1/1 (52.45% of suppression) up to 1/16 ratio (18.44% of suppression) for CD8^+^ T cells (Fig. 4d, e). Except 1/32 ratio in CD8 group, TNFα priming significantly increased CB-ECFC immunosuppressive capacity in all ratios of CD4^+^ (81.33% to 9.66% of suppression for 1/1 and 1/32 ratio, respectively) and CD8^+^ T cells (61.60% to 31.32% of suppression for 1/1 and 1/16 ratio, respectively) (Fig. 4d, e). Hence, we report an increased immunosuppressive effect of TNFα primed ECFCs which is more accentuated in CB-ECFCs than APB-ECFCs.

### Interaction of TNFα with TNFR2 and not TNFR1 increases ECFC immunosuppressive effect

**Fig. 5:**
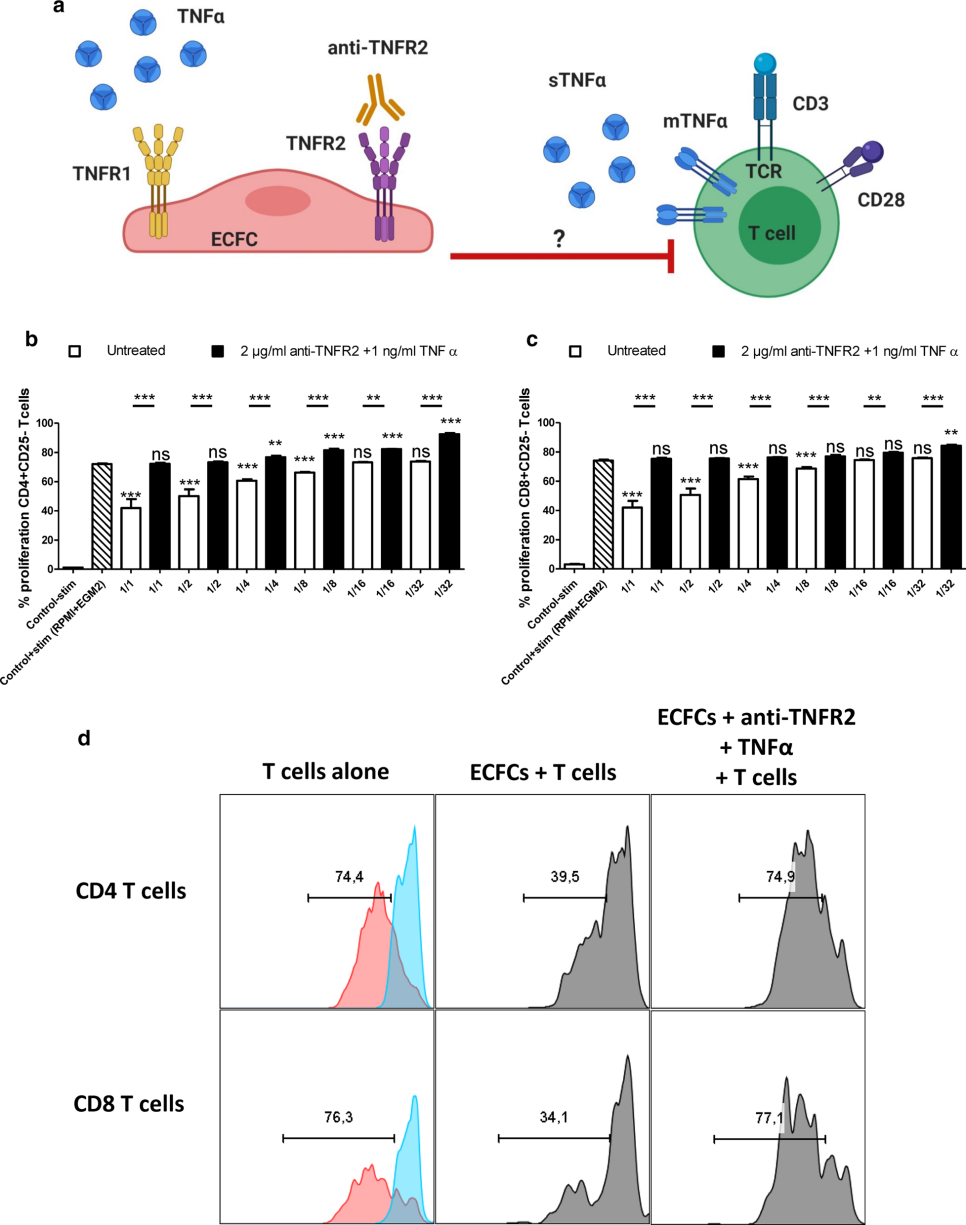
Primed EFCF’s increased immunosuppressive effect is TNFα-TNFR2 dependent. **a** This schematic depicts our hypothesis based on the immunogenic effect of TNFα treatment on ECFC while TNFR2 is blocked. Briefly, in the absence of TNFR2, treating ECFCs with TNFα could merely stimulate TNFR1. Therefore, we have hypothesized that in this setting, ECFCs may not properly exert their immunosuppressive effect. **b** ECFCs were treated with 2 µg/ml of anti-TNFR2 neutralizing Ab for 24 h followed by 1 ng/ml of TNFα for another 24 h. Activated CFSE^+^CD3^+^CD25^−^ responder T cells were co-cultured with CB-ECFCs in different ECFC/T cell ratios (n = 8). Proliferation of CD4^+^ T cells (**b**) and CD8^+^ T cells (**c**) was measured by flow cytometry. The first bar represents the unstimulated T cells alone, the second bar represents the anti-CD3/CD28 stimulated T cells alone in 50% RPMI + 50% EGM2 media. **d** A flow cytometry representative of proliferation assay at 1:1 ECFC to T cell ratio. Un-stimulated T cells alone are depicted in blue and their activated counterparts are depicted in red. Each histogram bar represents the percent of dividing cells. Data are represented as mean value ± SEM collected from 2 different experiments. One way ANOVA or Student *t* test analysis was performed to generate *P* values. ns: non-significant, *P < .05; **P < .01; ***P < .001. Stim: Anti-CD3/CD28 activation Beads

We next sought to understand which TNFR is responsible for increased ECFC immunosuppressive effect. Thus, we have re-created ECFC/T cell co-culture experiment but to selectively stimulate TNFR1, we blocked TNFR2 on ECFCs using anti-TNFR2 neutralizing Ab for 24 h. Then, we treated them with 1 ng/ml of TNFα for another 24 h and assessed their immunosuppressive effect (Fig. 5a). Untreated or primed CB-ECFCs were co-cultured with CFSE labeled CD3^+^CD25^−^ responder T cells in 6 increasing ratios (1/1 to 1/32 for ECFCs/T cells). After 3 days, T cells were collected and the proliferation capacity of CD4^+^ and CD8^+^ T cells was measured. Our result revealed that, while untreated CB-ECFCs suppressed T cells in a dose dependent manner, TNFR1-stimulated ECFCs did not exert any immunosuppressive effect regardless of ECFC/ T cell ratios (Fig. 5b–d). Surprisingly, TNFR1 stimulation directed ECFCs toward immunogenic phenotype as starting from 1/4 ratio, CD4^+^ T cells proliferated even more than activated T cells alone control group. This immunogenic effect was observed only in 1/32 ratio for CD8^+^ T cells (Fig. 5b, c). Altogether, these data proved that interaction of TNFα merely by TNFR2 is responsible of increased ECFC immunosuppressive effect.

### TNFα priming increases ECFC capacity to down-modulate T cell activation markers

**Fig. 6:**
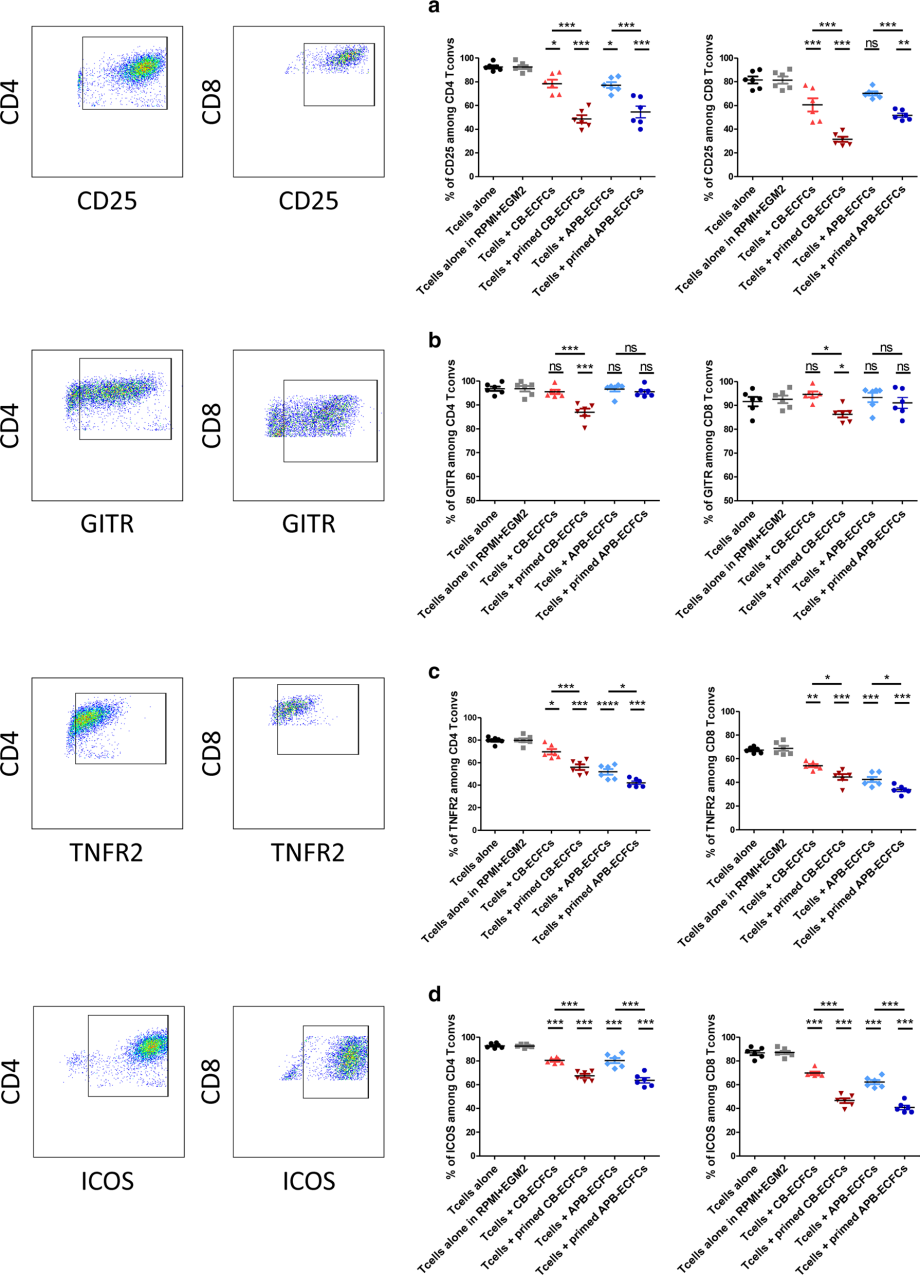
TNFα priming enhances ECFC immunomodulatory function. Anti-CD3/CD28 activated human CD3^+^CD25^−^ T cells were co-cultured with CB-ECFCs and APB-ECFCs in a fixed 1:6 ECFC to T cell ratio. After 3 day, T cells were collected and activation markers (CD25, GITR, TNFR2 and ICOS) were analysed by flow cytometry. Representative flow cytometry dot plots show the percentage of CD25, GITR, TNFR2 and ICOS among CD4^+^ T cells from the activated T cells control group (left panel) and among CD8^+^ T cells from the activated T cells control group (right panel). Frames defined the positive subpopulations for each marker analysis in the CD4^+^ and CD8^+^ population. Each dot represents a measured value collected from 2 different experiments (n = 6). For each group of values, horizontal lines represent mean value ± SEM. One way ANOVA analysis was performed to generate *P* values. ns: non-significant, *P < .05; **P < .01; ***P < .001. T convs: conventional T cells

To understand if TNFα priming increases ECFC capacity to modulate T cell activation profile, CB-ECFCs and APB-ECFCs were co-cultured with activated CD3^+^CD25^−^ T cells at a fixed 1:6 ECFC to T cell ratio. After 3 days, T cells were collected and analyzed for the percentage of expression and the MFI of different activation markers among CD4^+^ and CD8^+^ T cells. We first measured the expression of CD25 marker which is constitutively expressed on T reg and activated T cell [52, 53]. We observed a dramatic reduction of CD25 expression among CD4^+^ T cells after co-culturing with CB-ECFCs or APB-ECFCs and among CD8^+^ T cells while co-cultured with CB-ECFCs (Fig. 6a and Additional file 1: Figure S1). Priming CB-ECFCs and APB-ECFCs with 1 ng/ml of TNFα significantly increased their immunomodulatory effect against both T cell populations (Fig. 6a and Additional file 1: Figure S1). Moreover, we evaluated the expression of two members of TNFα receptor superfamily, GITR (TNFRSF18) and TNFR2 (TNFRSF1B). Our results showed that while CB-ECFCs were able to down-modulate only the MFI of GITR on CD4^+^ T cells, TNFα priming of CB-ECFCs remarkably reduced the percentage of expression and MFI of GITR among CD4^+^ and CD8^+^ T cells (Fig. 6b and Additional file 1: Figure S1). Interestingly, even though APB-ECFCs were unable to decrease the percentage and MFI of GITR in neither of T cell populations, TNFα priming of APB-ECFCs led to a significant reduction in the MFI of GITR on CD4^+^ T cells (Additional file 1: Figure S1). In case of TNFR2 marker, we noticed that while both CB-ECFCs and APB-ECFCs were able to significantly decrease the TNFR2 expression, APB-ECFCs demonstrated a stronger modulatory effect. Once more, priming ECFCs with 1 ng/ml of TNFα increased ECFC immunomodulatory effect regardless of the ECFC source or T cell population (Fig. 6c). Furthermore, except an increased expression level on CD4^+^ T cells co-cultured with primed APB-ECFCs, our result did not reveal any alteration in the MFI of TNFR2 on neither of T cell populations (Additional file 1: Figure S1). Finally, we studied the expression of inducible co-stimulatory molecule (ICOS). This co-stimulatory receptor is essential for T cell activation and proliferation [54]. We observed a significant reduction of ICOS expression among CD4^+^ and CD8^+^ T cells co-cultured with either of ECFCs (Fig. 6d and Additional file 1: Figure S1). Once again, TNFα priming led to increased ECFC immunomodulatory effect (Fig. 6d and Additional file 1: Figure S1). Altogether, these data suggest that priming ECFCs with TNFα can strongly increase their immunomodulatory effect.

## Discussion

Certain cardiovascular and pulmonary disorders are linked with inefficiency and paucity of circulating EPCs [9, 55–58]. It has been evidenced that endothelial dysfunction could negatively impacts cardiac function, heart failure progression, and survival [59, 60]. Transplantations are other conditions that EPC contribution to vessel formation is crucial. Infusion of EPCs was shown to accelerate hematopoietic and immune reconstitution, restoration of vascular niche in BM and ameliorate GVHD after hematopoietic stem cell transplantation (HSCT) mostly via improving the integrity of sinusoidal endothelial cells of the BM [61–65]. Interestingly, administration of anti-vascular endothelial cadherin antibody significantly disrupted these protective effects [62]. In case of solid transplantation, long-term survival and eventual acceptance of transplants has been also associated to correct and rapid revascularization [66, 67].

Due to their special regenerative and immunologic properties, EPCs are ideal cell therapy candidates in conditions that both angiogenesis and immunomodulation are involved. Since EPCs from patients are usually dysfunctional or/and exist in low numbers, allogenic sources of EPCs especially from CB are good alternatives. Compared to APB-EPCs, CB-EPCs are more clonogenic, efficient and immunotolerated [17, 18, 28]. Nevertheless, the choice of allogenic sources usually raise the concern of immune rejection risking the achievement of the desired benefit.

We have recently demonstrated that EPCs bear immunosuppressive effect, secrete anti-inflammatory cytokines and are tolerated for certain days after their administration in xenogeneic ischemia model [28, 29]. However, our experience demonstrates that compared to other immunosuppressive cells like Tregs and MSCs, EPCs are significantly less suppressive. This could potentially affect the success of cell therapy outcome. Furthermore, it has been evidenced that compared to fast ingrowth of recipient vasculature, the contribution of EPCs to revascularization begins after 7 days [66]. This highlights the importance of finding a solution to increase their immunosuppressive and immunoregulatory functions leading to their longer-lasting presence *in-vivo* after their allogenic transplantation.

Several studies reported that immunosuppressive effect of verity of cells including Tregs, B regs, MDSCs and MSCs is TNFR2 dependent [44, 49, 50, 68, 69]. Our recent results proved that ECFCs are also sharing the same mechanism [28]. Indeed, inflammatory factors, like IL-1, TNFα and IFNγ, enhance MSC and Treg immunoregulatory functions mostly through promoting the secretion of immunoregulatory factors like PGE2, IDO, HGF, IL-10 and TGFβ [43, 52, 70–73]. Therefore, the main purpose of our study was to up-regulate TNFR2 immune checkpoint molecule in ECFCs via pre-treating them with a proper dose of TNFα and prime them towards the accentuated immunosuppressive effect.

We examined different doses of TNFα to find out the one wherein the desired inflammatory environment is preserved but does not cause the overexpression of the endothelial inflammatory-related injury markers such as ICAM, VCAM, and TIE-2 and ultimately elevated percentage of apoptotic cells. Our result proved that 1 ng/ml of TNFα affords the majority of those requirements. Indeed, we remarked using low TNFα doses (0.01 and 0.1 ng/ml) and high doses (50 and 100 ng/ml) led to higher expression of TNFR1 and elevated percentage of apoptotic cells without affecting TNFR2. This can be explained by the fact that unlike transmembrane form of TNFα (mTNFα) with higher affinity to TNFR2, its soluble form (sTNFα) is more affine to TNFR1 [74, 75]. Therefore, in low doses it preferably goes to TNFR1. In intermediate doses, after interacting with TNFR1, TNFα could stimulate TNFR2 and in higher doses, due to over saturation, the excess TNFα turns once again over TNFR1. This demonstrates that respecting the correct dose is extremely important and could radically change the outcome of priming process. Here, to be closer to normal physiological conditions, we have used TNFα priming but we are conscious that utilizing TNFR2 agonist antibodies to specifically stimulate this receptor may be a better approach.

It would be unreasonable to believe that treating ECFCs with 1 ng/ml of TNFα does not provoke TNFR1 signaling, even though it does not lead to its up-regulation. Increased percentage of apoptotic cells treated with 1 ng/ml of TNFα is an evidence for TNFR1 stimulation. Therefore, there must be a sort of crosstalk between the two receptors. Despite having two distinct actions, both receptors can activate NF-κB signaling pathway through TNF receptor associated factors (TRAFs) involvement [76]. It is demonstrated that TNFR family co-stimulation increases Treg activation and function via NF-κB [77, 78]. This could explain why priming ECFCs with 1 ng/ml of TNFα could still increase ECFC immunoregulatory effect. Apparently, this cross talk might be in an undemocratic manner since TNFR2 seems to govern the final fate of TNFα signaling cascade.

After selecting the proper dose, we have pre-treated two available sources of ECFCs (APB and CB) with 1 ng/ml of TNFα for duration of 24 h. This incubation time has been chosen in respect to augmented apoptosis observed after 48 h of incubation (data not shown). As expected, pre-treatment of ECFCs significantly increased their immunosuppressive and immunomodulatory effect against fully-HLA-mismatched allogenic T cells. This was proven by a decreased T cells proliferation capacity and down-modulation of T cell activation markers. Indeed, this effect was TNFR2 dependent since after blocking this receptor not only we did not observe any immunosuppression but also a dose dependent immunogenic function evidenced by extensive T cell proliferation.

The up-regulation of TNFR2 on immunosuppressive cells is already related to their increased capacity of IL-10 and TGFβ secretion [50, 79]. Accordingly, adding 1 ng/ml of TNFα significantly increased the production of IL-10, TGFβ and HLA-G anti-inflammatory cytokines by ECFCs [28]. Interestingly, this was also TNFR2 dependent since blocking TNFR2 led to their dramatic decrease even bellow their basal secretion level [28]. In contrary, up-regulation of TNFR1 was associated to increased pro-inflammatory cytokine secretion [75, 80]. Although this could be potentially the reason for increased ECFC immunosuppressive effect, further experiments including pro-inflammatory cytokine measurement (after TNFR2 blocking) and blocking anti-inflammatory cytokines is necessary.

Previous studies on EPCs have reported that TNFα-TNFR1 signaling increases deleterious mechanisms and TNFα-TNFR2 signaling supports the pro-angiogenic and protective ones [41, 42, 45–48, 81]. Correspondingly, we report for the first time that TNFα-TNFR1 axis increases immunogenic and TNFα-TNFR2 increases the immunosuppressive EPC properties.

In the future it will be necessary to prime ECFCs either by TNFα or more accurately by TNFR2 agonist to take advantage of their boosted pro-angiogenic and immunosuppressive effect and evaluate their efficiency in an *in-vivo* ischemia or transplant model. It will be also interesting to investigate if EPC priming could restore their diminished function especially in adult patients with cardiovascular disorders.

## Conclusion

We believe that our work brings some understanding to the complex crosstalk between T cells and EPCs in inflammatory conditions and demonstrates the crucial role of TNFα-TNFR2 signaling pathway in EPC immunoregulatory functions. TNFα priming of EPCs could provide a new tool to ameliorate EPC therapy through enhancing their immunosuppressive and angiogenic properties. This could allow using reduced cell numbers with increased efficiency to prevent and treat patients with cardiovascular disorders.

## Supplementary Information

**Additional file 1.** **Supplementary Figure 1: TNFα priming enhances ECFC capacity to down-modulate T cell activation markers.** Anti-CD3/CD28 activated human CD3^+^CD25^−^ T cells were co-cultured with CB-ECFCs and APB-ECFCs in a fixed 1:6 ECFC to T cell ratio. After 3 day, T cells were collected and the MFI of activation markers (CD25, GITR, TNFR2 and ICOS) were analysed by flow cytometry. The markers were studied among CD4^+^ Tconvs (left graphs) and among CD8^+^ Tconvs (right graphs). MFI values have been normalized with T cells alone control group. Each dot represents a measured value collected from 2 different experiments (n=6). For each group of values, horizontal lines represent mean value ± SEM. One way ANOVA analysis was performed to generate P values.

## Data Availability

The datasets used and/or analysed during the current study are available from the corresponding author on reasonable request.

## Abbreviations

APB: Adult Peripheral Blood
B reg: Regulatory B cell
bFGF: Basic Fibroblast Growth Factor
BM: Bone Marrow
CB: Cord Blood
CFSE: Carboxyfluorescein succinimidyl ester
CTEPH: Chronic Thromboembolic Pulmonary Hypertension
CVD: Cardiovascular Disorders
ECFCs: Endothelial Colony Forming Cells
ECs: Endothelial Cells
EPCs: Endothelial Progenitor Cells
GVHD: Graft Versus Host Disease
HSCT: Hematopoietic Stem Cell Transplantation
ICOS: Inducible Co-Stimulatory molecule
mAb: Monoclonal Anti Body
MACS: Magnetic-Activated Cell Sorting
MDSCs: Myeloid Derived Suppressive Cells
MFI: Mean Fluorescence Intensity
MNC: Mononuclear cells
MSCs: Mesenchymal Stem Cells
P: Passage
T conv: Conventional T cell
T reg: Regulatory T cell
TNFR1: Tumor Necrosis Factor Receptor 1
TNFR2: Tumor Necrosis Factor Receptor 2
TNFα: Tumor Necrosis Factor alpha
VEGF: Vascular Endothelial Growth Factor
VEGFR2: Vascular Endothelial Growth Factor Receptor 2

## References

[1] Widmer RJ, Lerman A (2014). Endothelial dysfunction and cardiovascular disease. Glob Cardiol Sci Pract.

[2] Carmeliet P, Jain RK (2011). Molecular mechanisms and clinical applications of angiogenesis. Nature.

[3] Losordo DW, Dimmeler S (2004). Therapeutic angiogenesis and vasculogenesis for ischemic disease. Part I: angiogenic cytokines. Circulation.

[4] Deveza L, Choi J, Yang F (2012). Therapeutic angiogenesis for treating cardiovascular diseases. Theranostics.

[5] Tanaka M, Taketomi K, Yonemitsu Y (2014). Therapeutic angiogenesis: recent and future prospects of gene therapy in peripheral artery disease. Curr Gene Ther.

[6] Hooper AT, Butler JM, Nolan DJ (2009). Engraftment and reconstitution of hematopoiesis is dependent on VEGFR2-mediated regeneration of sinusoidal endothelial cells. Cell Stem Cell.

[7] Osipova O, Saaya S, Karpenko A, Zakian S, Aboian E (2019). Cell therapy of critical limb ischemia-problems and prospects. VASA Z Gefasskrankh.

[8] Qadura M, Terenzi DC, Verma S, Al-Omran M, Hess DA (2018). Concise review: cell therapy for critical limb ischemia: an integrated review of preclinical and clinical studies. Stem Cells Dayt Ohio.

[9] Loisel F, Provost B, Guihaire J (2019). Autologous endothelial progenitor cell therapy improves right ventricular function in a model of chronic thromboembolic pulmonary hypertension. J Thorac Cardiovasc Surg.

[10] Asahara T, Murohara T, Sullivan A (1997). Isolation of putative progenitor endothelial cells for angiogenesis. Science.

[11] Masuda H, Asahara T (2003). Post-natal endothelial progenitor cells for neovascularization in tissue regeneration. Cardiovasc Res.

[12] Wang T, Fang X, Yin Z-S (2018). Endothelial progenitor cell-conditioned medium promotes angiogenesis and is neuroprotective after spinal cord injury. Neural Regen Res.

[13] Takahashi T, Kalka C, Masuda H (1999). Ischemia- and cytokine-induced mobilization of bone marrow-derived endothelial progenitor cells for neovascularization. Nat Med.

[14] Walter DH, Rittig K, Bahlmann FH (2002). Statin therapy accelerates reendothelialization: a novel effect involving mobilization and incorporation of bone marrow-derived endothelial progenitor cells. Circulation.

[15] Kalka C, Masuda H, Takahashi T (2000). Transplantation of ex vivo expanded endothelial progenitor cells for therapeutic neovascularization. Proc Natl Acad Sci U S A.

[16] Uzan G, Vanneaux V, Delmau C, Ayoubi F, Gluckman E, Larghero J (2009). Cord blood circulating endothelial progenitors: perspectives for clinical use in cardiovascular diseases. Bull Acad Natl Med.

[17] Ferratge S, Ha G, Carpentier G (2017). Initial clonogenic potential of human endothelial progenitor cells is predictive of their further properties and establishes a functional hierarchy related to immaturity. Stem Cell Res.

[18] Ha G, Ferratge S, Naserian S (2018). Circulating endothelial progenitors in vascular repair. J Cell Immunother.

[19] Ha G, De Torres F, Arouche N (2019). GDF15 secreted by senescent endothelial cells improves vascular progenitor cell functions. PLoS ONE.

[20] Yoder MC, Mead LE, Prater D (2007). Redefining endothelial progenitor cells via clonal analysis and hematopoietic stem/progenitor cell principals. Blood.

[21] Sieveking DP, Buckle A, Celermajer DS, Ng MKC (2008). Strikingly different angiogenic properties of endothelial progenitor cell subpopulations: insights from a novel human angiogenesis assay. J Am Coll Cardiol.

[22] Au P, Daheron LM, Duda DG (2008). Differential in vivo potential of endothelial progenitor cells from human umbilical cord blood and adult peripheral blood to form functional long-lasting vessels. Blood.

[23] Pearson JD (2010). Endothelial progenitor cells: an evolving story. Microvasc Res.

[24] Yoder MC (2012). Human endothelial progenitor cells. Cold Spring Harb Perspect Med.

[25] Guillevic O, Ferratge S, Pascaud J, Driancourt C, Boyer-Di-Ponio J, Uzan G (2016). A novel molecular and functional stemness signature assessing human cord blood-derived endothelial progenitor cell immaturity. PLoS ONE.

[26] Boyer-Di Ponio J, El-Ayoubi F, Glacial F (2014). Instruction of circulating endothelial progenitors in vitro towards specialized blood-brain barrier and arterial phenotypes. PLoS ONE.

[27] Naserian S, Abdelgawad ME, Lachaux J (2019). Development of bio-artificial micro-vessels with immunosuppressive capacities: a hope for future transplantations and organoids. Blood.

[28] Naserian S, Abdelgawad ME, Afshar Bakshloo M (2020). The TNF/TNFR2 signaling pathway is a key regulatory factor in endothelial progenitor cell immunosuppressive effect. Cell Commun Signal CCS.

[29] Proust R, Ponsen A-C, Rouffiac V (2020). Cord blood-endothelial colony forming cells are immunotolerated and participate at post-ischemic angiogenesis in an original dorsal chamber immunocompetent mouse model. Stem Cell Res Ther.

[30] Prisco AR, Hoffmann BR, Kaczorowski CC (2016). Tumor Necrosis Factor α Regulates Endothelial Progenitor Cell Migration via CADM1 and NF-kB. Stem Cells Dayt Ohio.

[31] Leibovich SJ, Polverini PJ, Shepard HM, Wiseman DM, Shively V, Nuseir N (1987). Macrophage-induced angiogenesis is mediated by tumour necrosis factor-alpha. Nature.

[32] Sato N, Fukuda K, Nariuchi H, Sagara N (1987). Tumor necrosis factor inhibiting angiogenesis in vitro. J Natl Cancer Inst.

[33] Fràter-Schröder M, Risau W, Hallmann R, Gautschi P, Böhlen P (1987). Tumor necrosis factor type alpha, a potent inhibitor of endothelial cell growth in vitro, is angiogenic in vivo. Proc Natl Acad Sci U S A.

[34] Fajardo LF, Kwan HH, Kowalski J, Prionas SD, Allison AC (1992). Dual role of tumor necrosis factor-alpha in angiogenesis. Am J Pathol.

[35] Salomon BL, Leclerc M, Tosello J, Ronin E, Piaggio E, Cohen JL (2018). Tumor necrosis factor α and regulatory T cells in oncoimmunology. Front Immunol.

[36] Yang S, Wang J, Brand DD, Zheng SG (2018). Role of TNF-TNF receptor 2 signal in regulatory T cells and its therapeutic implications. Front Immunol.

[37] Yan L, Zheng D, Xu R-H (2018). Critical role of tumor necrosis factor signaling in mesenchymal stem cell-based therapy for autoimmune and inflammatory diseases. Front Immunol.

[38] Yoshida S, Ono M, Shono T (1997). Involvement of interleukin-8, vascular endothelial growth factor, and basic fibroblast growth factor in tumor necrosis factor alpha-dependent angiogenesis. Mol Cell Biol.

[39] Krönke M, Schütze S, Scheurich P, Pfizenmaier K (1992). TNF signal transduction and TNF-responsive genes. Immunol Ser.

[40] Hoefer IE, van Royen N, Rectenwald JE (2002). Direct evidence for tumor necrosis factor-alpha signaling in arteriogenesis. Circulation.

[41] Monden Y, Kubota T, Inoue T (2007). Tumor necrosis factor-alpha is toxic via receptor 1 and protective via receptor 2 in a murine model of myocardial infarction. Am J Physiol Heart Circ Physiol.

[42] Kishore R, Tkebuchava T, Sasi SP (2011). Tumor necrosis factor-α signaling via TNFR1/p55 is deleterious whereas TNFR2/p75 signaling is protective in adult infarct myocardium. Adv Exp Med Biol.

[43] Pierini A, Strober W, Moffett C (2016). TNF-α priming enhances CD4+FoxP3+ regulatory T-cell suppressive function in murine GVHD prevention and treatment. Blood.

[44] Leclerc M, Naserian S, Pilon C (2016). Control of GVHD by regulatory T cells depends on TNF produced by T cells and TNFR2 expressed by regulatory T cells. Blood.

[45] Katare RG, Ando M, Kakinuma Y, Arikawa M, Yamasaki F, Sato T (2010). Differential regulation of TNF receptors by vagal nerve stimulation protects heart against acute ischemic injury. J Mol Cell Cardiol.

[46] Aggarwal S, Gollapudi S, Gupta S (1999). Increased TNF-alpha-induced apoptosis in lymphocytes from aged humans: changes in TNF-alpha receptor expression and activation of caspases. J Immunol Baltim Md 1950.

[47] Goukassian DA, Qin G, Dolan C (2007). Tumor necrosis factor-alpha receptor p75 is required in ischemia-induced neovascularization. Circulation.

[48] Luo Y, Xu Z, Wan T (2010). Endothelial-specific transgenesis of TNFR2 promotes adaptive arteriogenesis and angiogenesis. Arterioscler Thromb Vasc Biol.

[49] Polz J, Remke A, Weber S (2014). Myeloid suppressor cells require membrane TNFR2 expression for suppressive activity. Immun Inflamm Dis.

[50] Ticha O, Moos L, Wajant H, Bekeredjian-Ding I (2017). Expression of tumor necrosis factor receptor 2 characterizes TLR9-driven formation of interleukin-10-producing B cells. Front Immunol.

[51] Sierra-Parraga JM, Merino A, Eijken M (2020). Reparative effect of mesenchymal stromal cells on endothelial cells after hypoxic and inflammatory injury. Stem Cell Res Ther.

[52] Singh B, Read S, Asseman C (2001). Control of intestinal inflammation by regulatory T cells. Immunol Rev.

[53] Reddy M, Eirikis E, Davis C, Davis HM, Prabhakar U (2004). Comparative analysis of lymphocyte activation marker expression and cytokine secretion profile in stimulated human peripheral blood mononuclear cell cultures: an in vitro model to monitor cellular immune function. J Immunol Methods.

[54] Dong C, Juedes AE, Temann UA (2001). ICOS co-stimulatory receptor is essential for T-cell activation and function. Nature.

[55] Werner N, Kosiol S, Schiegl T (2005). Circulating endothelial progenitor cells and cardiovascular outcomes. N Engl J Med.

[56] Schmidt-Lucke C, Rössig L, Fichtlscherer S (2005). Reduced number of circulating endothelial progenitor cells predicts future cardiovascular events: proof of concept for the clinical importance of endogenous vascular repair. Circulation.

[57] Cristóvão G, Milner J, Sousa P (2020). Improvement in circulating endothelial progenitor cells pool after cardiac resynchronization therapy: increasing the list of benefits. Stem Cell Res Ther.

[58] Huertas A, Palange P (2011). Circulating endothelial progenitor cells and chronic pulmonary diseases. Eur Respir J.

[59] Fischer D, Rossa S, Landmesser U (2005). Endothelial dysfunction in patients with chronic heart failure is independently associated with increased incidence of hospitalization, cardiac transplantation, or death. Eur Heart J.

[60] Nonaka-Sarukawa M, Yamamoto K, Aoki H (2007). Circulating endothelial progenitor cells in congestive heart failure. Int J Cardiol.

[61] Zeng L, Chen C, Song G (2012). Infusion of endothelial progenitor cells accelerates hematopoietic and immune reconstitution, and ameliorates the graft-versus-host disease after hematopoietic stem cell transplantation. Cell Biochem Biophys.

[62] Yan Z, Zeng L, Li Z (2013). Bone marrow-derived endothelial progenitor cells promote hematopoietic reconstitution after hematopoietic stem cell transplantation. Transplant Proc.

[63] Khoo CP, Pozzilli P, Alison MR (2008). Endothelial progenitor cells and their potential therapeutic applications. Regen Med.

[64] Zhang Y, Song G, Pan B, Hua J, Xu K, Zeng L (2012). Recovery of vascular niche in bone marrow by donor derived endothelial progenitor cells after allogeneic bone marrow transplantation in mice. Zhonghua Xue Ye Xue Za Zhi Zhonghua Xueyexue Zazhi.

[65] Salter AB, Meadows SK, Muramoto GG (2009). Endothelial progenitor cell infusion induces hematopoietic stem cell reconstitution in vivo. Blood.

[66] Capla JM, Ceradini DJ, Tepper OM (2006). Skin graft vascularization involves precisely regulated regression and replacement of endothelial cells through both angiogenesis and vasculogenesis. Plast Reconstr Surg.

[67] Geeroms M, Hamdi M, Hirano R (2019). Quality and quantity-cultured murine endothelial progenitor cells increase vascularization and decrease fibrosis in the fat graft. Plast Reconstr Surg.

[68] Beldi G, Khosravi M, Abdelgawad ME (2020). TNFα/TNFR2 signaling pathway: an active immune checkpoint for mesenchymal stem cell immunoregulatory function. Stem Cell Res Ther.

[69] Beldi G, Bahiraii S, Lezin C, Nouri Barkestani M, Abdelgawad ME, Uzan G, Naserian S. TNFR2 is a crucial hub controlling mesenchymal stem cell biological and functional properties. Front. Cell Dev Biol. 2020. 10.3389/fcell.2020.596831. 10.3389/fcell.2020.596831 PMC7746825 33344453

[70] Yang H-M, Song W-J, Li Q (2018). Canine mesenchymal stem cells treated with TNF-α and IFN-γ enhance anti-inflammatory effects through the COX-2/PGE2 pathway. Res Vet Sci.

[71] Kim DS, Jang IK, Lee MW (2018). Enhanced immunosuppressive properties of human mesenchymal stem cells primed by interferon-γ. EBioMedicine.

[72] Redondo-Castro E, Cunningham C, Miller J (2017). Interleukin-1 primes human mesenchymal stem cells towards an anti-inflammatory and pro-trophic phenotype in vitro. Stem Cell Res Ther.

[73] Prasanna SJ, Gopalakrishnan D, Shankar SR, Vasandan AB (2010). Pro-inflammatory cytokines, IFNγ and TNFα, influence immune properties of human bone marrow and Wharton jelly mesenchymal stem cells differentially. PLoS ONE.

[74] Grell M, Douni E, Wajant H (1995). The transmembrane form of tumor necrosis factor is the prime activating ligand of the 80 kDa tumor necrosis factor receptor. Cell.

[75] Wajant H, Siegmund D (2019). TNFR1 and TNFR2 in the Control of the life and death balance of macrophages. Front Cell Dev Biol.

[76] Naudé PJW, den Boer JA, Luiten PGM, Eisel ULM (2011). Tumor necrosis factor receptor cross-talk. FEBS J.

[77] Lubrano di Ricco M, Ronin E, Collares D (2020). Tumor necrosis factor receptor family costimulation increases regulatory T-cell activation and function via NF-κB. Eur J Immunol.

[78] Ronin E, Lubrano di Ricco M, Vallion R (2019). The NF-κB RelA transcription factor is critical for regulatory T cell activation and stability. Front Immunol.

[79] Hu X, Li B, Li X (2014). Transmembrane TNF-α promotes suppressive activities of myeloid-derived suppressor cells via TNFR2. J Immunol Baltim Md 1950.

[80] Kuno R, Wang J, Kawanokuchi J, Takeuchi H, Mizuno T, Suzumura A (2005). Autocrine activation of microglia by tumor necrosis factor-alpha. J Neuroimmunol.

[81] Zhang Y, Zhao J, Lau WB (2013). Tumor necrosis factor-α and lymphotoxin-α mediate myocardial ischemic injury via TNF receptor 1, but are cardioprotective when activating TNF receptor 2. PLoS ONE.

