# Supplementary material for: TNFα priming through its interaction with TNFR2 enhances endothelial progenitor cell immunosuppressive effect: new hope for their widespread clinical application
Source: Cell Commun Signal. 2021 Jan 4;19:1. doi: 10.1186/s12964-020-00683-x (PMC7784277; doi:10.1186/s12964-020-00683-x)
Supplement: Supplementary file 2 — Additional file 1. Supplementary Figure 1: TNFα priming enhances ECFC capacity to down-modulate T cell activation markers. Anti-CD3/CD28 activated human CD3+CD25− T cells were co-cultured with CB-ECFCs and APB-ECFCs in a fixed 1:6 ECFC to T cell ratio. After 3 day, T cells were collected and the MFI of activation markers (CD25, GITR, TNFR2 and ICOS) were analysed by flow cytometry. The markers were studied among CD4+ Tconvs (left graphs) and among CD8+ Tconvs (right graphs). MFI values have been normalized with T cells alone control group. Each dot represents a measured value collected from 2 different experiments (n=6). For each group of values, horizontal lines represent mean value ± SEM. One way ANOVA analysis was performed to generate P values. [file 12964_2020_683_MOESM2_ESM.docx]

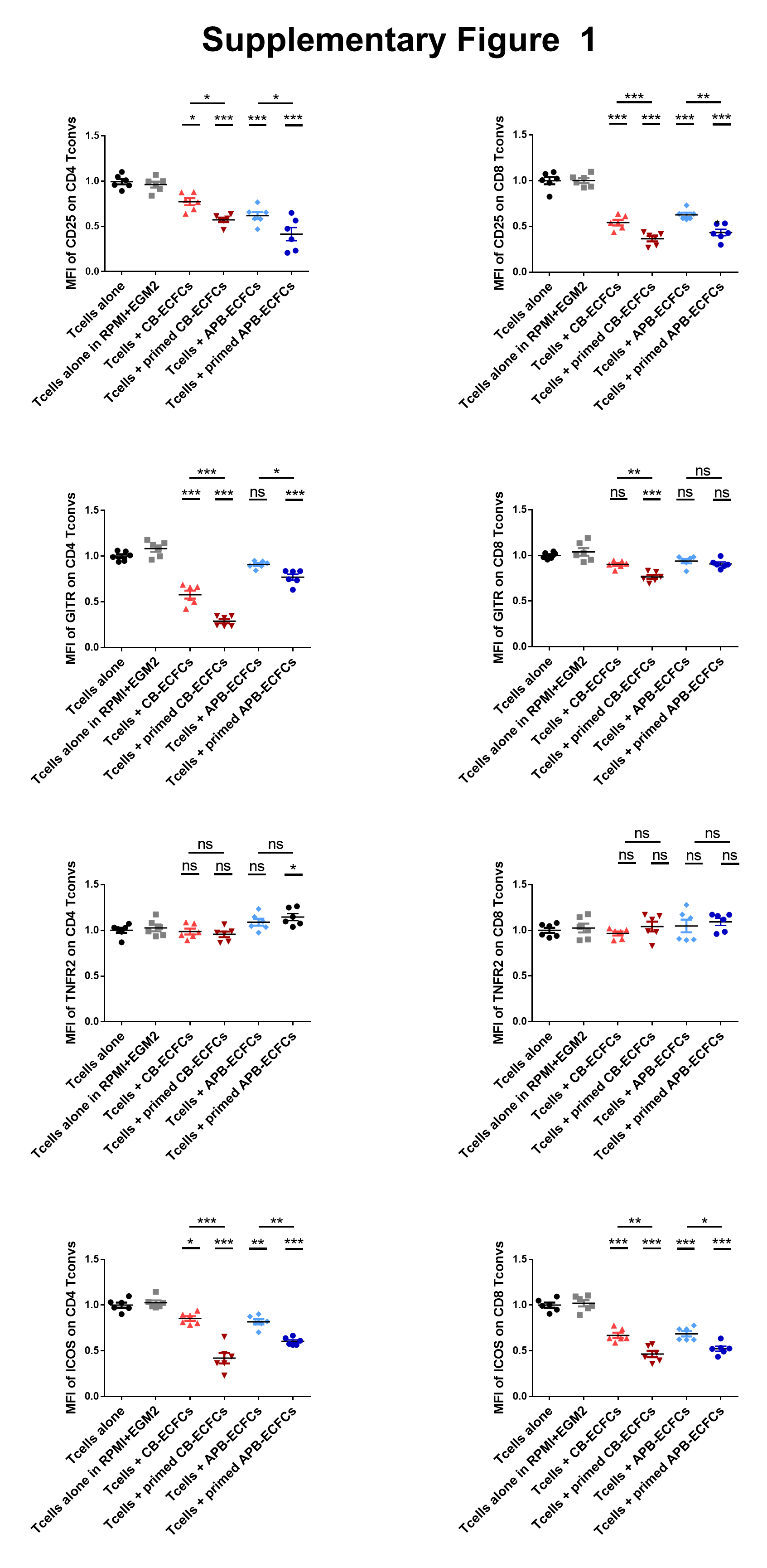


**Additional file 1: Figure S1.** TNFα priming enhances ECFC capacity to down-modulate T cell activation markers. Anti-CD3/CD28 activated human CD3^+^CD25^-^ T cells were co-cultured with CB-ECFCs and APB-ECFCs in a fixed 1:6 ECFC to T cell ratio. After 3 day, T cells were collected and the MFI of activation markers (CD25, GITR, TNFR2 and ICOS) were analysed by flow cytometry. The markers were studied among CD4^+^ Tconvs (left graphs) and among CD8^+^ Tconvs (right graphs). MFI values have been normalized with T cells alone control group. Each dot represents a measured value collected from 2 different experiments (n=6). For each group of values, horizontal lines represent mean value ± SEM. One way ANOVA analysis was performed to generate P values. ns: non-significant, *P<.05, **P<.01, ***P<.001. T convs: conventional T cells, MFI: Mean Fluorescence Intensity.
